# Basic Operative Tactics for Pulmonary Echinococcosis in the Era of Endostaplers and Energy Devices

**DOI:** 10.3390/medicina59030543

**Published:** 2023-03-10

**Authors:** Estera Bakinowska, Konstantinos Kostopanagiotou, Małgorzata Edyta Wojtyś, Kajetan Kiełbowski, Konrad Ptaszyński, Darko Gajić, Nikola Ruszel, Janusz Wójcik, Tomasz Grodzki, Periklis Tomos

**Affiliations:** 1 Department of Thoracic Surgery and Transplantation, Pomeranian Medical University in Szczecin, Alfreda Sokołowskiego 11, 70-891 Szczecin, Poland; 2 Department of Thoracic Surgery, Attikon University Hospital, National and Kapodistrian University of Athens, Rimini 1, 12462 Athens, Greece; 3 Department of Pathology, Faculty of Medicine, Collegium Medicum, University of Warmia and Mazury in Olsztyn, 10-561 Olsztyn, Poland; 4 Department of Internal Medicine and Hypertension with Subdepartment of Cardiology, Independent Public Provincial Hospital in Szczecin, Alfreda Sokołowskiego 11, 70-891 Szczecin, Poland

**Keywords:** echinococcosis, pulmonary echinococcosis, thoracic surgery

## Abstract

Human echinococcosis is a zoonotic infection caused by the larvae of the tapeworm species *Echinococcus.* The liver is the most common location for a primary echinococcosis. However, the parasite may bypass or spread from the liver to the lungs, causing primary or secondary pulmonary echinococcosis, respectively. Pulmonary echinococcosis is a clinically challenging condition in which anthelminthic regiments are important, but surgery has the central role in removing the cysts and preventing recurrences. Surgical treatment may involve cystotomy, enucleation, capitonnage, or atypical resections, which occasionally are in combination with hepatic procedures. The utilization of modern devices is greatly underdescribed in surgery for thoracic infections, even though these facilitate much of the work. Therefore, this article aims to describe pulmonary echinococcosis and the role of modern surgical devices in the treatment process. Furthermore, we report surgical treatment of three different cases of pulmonary echinococcosis. Surgeries of uncomplicated and ruptured hepatic or pulmonary cysts are described. Simple small pulmonary echinococcal lesions can be excised by endostaplers both for diagnostic and curative reasons. Larger cysts can be removed by energy devices unless large bronchial air leaks occur. Complicated cysts require treatment by more extensive techniques. Inexperienced surgeons should not abstain but should carefully decide preoperatively how to proceed.

## 1. Introduction

Human echinococcosis is a zoonotic infection caused by the larvae of the tapeworm species *Echinococcus* (*E.*). Two of the four known pathogenic species to humans, canine (*E. granulosus*) and fox tapeworm (*E. multilocularis*), cause cystic echinococcosis (CE) and alveolar echinococcosis (AE), respectively [1]. Humans become accidental hosts when they inhale or consume helminthic eggs from contaminated water and food. These enter portal circulation forming hepatic cysts, which is the commonest site for a primary echinococcosis. Pulmonary echinococcosis (PE) is a variant of the disease, in which parasites form lesions in the lungs. The pathogenesis of PE may involve bypassing the liver (primary PE) or metastatic-like spread from the liver (secondary PE) [2]. Primary or secondary PE is a clinically challenging condition in which anthelminthic regiments are important, but surgery has the central role in removing the cysts and preventing recurrences [3]. The aim of treatment is to completely remove the pulmonary cysts through delicate dissection, avoiding spillage of the highly infectious larvae. The majority of modern thoracic surgeons rarely come across PE, being unfamiliar with the various procedures used by older generations of surgeons. We consider though the modern surgical devices to simplify this complex task. To support this view, we go through some of the typical cases, providing tips for the inexperienced surgeons in this field for a successful management.

## 2. Case Presentations

### 2.1. Case 1

**Figure 1:**
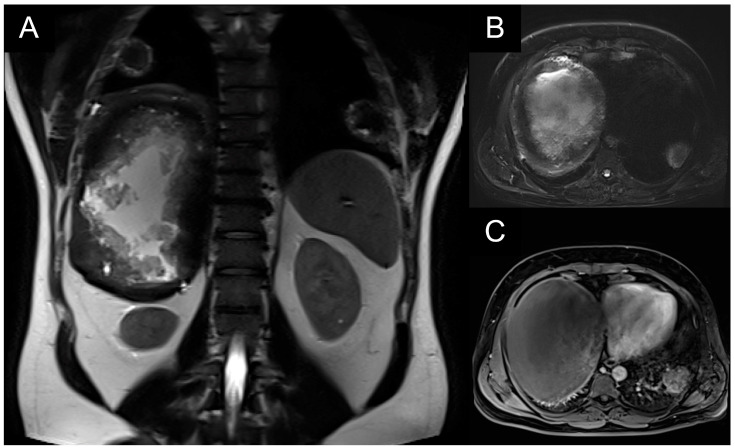
(**A**,**B**): T2-weighted hyperintense liver lesion; (**C**): T1-weighted hypointense liver lesion with abnormal pulmonary lesions.

**Figure 2:**
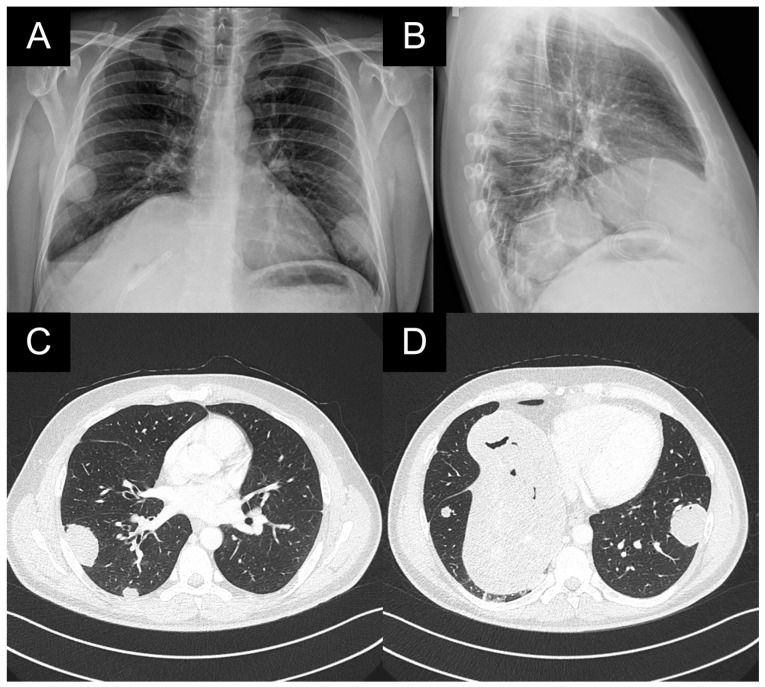
(**A**,**B**): Preoperative X-ray images of bilateral lesions in the lower lobes; (**C**): CT scan with the lesions in the right segments 6 and 8 measuring 4.6 and 1.4 cm; (**D**): 1 cm lesion in the 8th right segment and 4 cm lesion in the 9th left segment. An enlarged hepatic cyst percutaneously drained by hepatocentesis.

A patient with multiple bilateral lung lesions was diagnosed after investigations for a large hepatic lesion that turned out to be echinococcosis. A 41-year-old male patient reported abdominal pain and shortness of breath and was referred to the hospital by a primary care physician. Primary radiological investigations revealed a large hepatic lesion and multiple bilateral pulmonary lesions. Magnetic resonance imaging (MRI) was performed to evaluate the hepatic lesion (Figure 1), while chest X-rays and computed tomography (CT) were performed prior to admission to the Department of Thoracic Surgery. CT detected two right lower lobe lesions in segment 6 (diameters 4.6 cm, 1.4 cm) and one in segment 8 measuring 1 cm. In the left lower lobe, there was a single 4 cm lesion in segment 9 (Figure 2).

**Figure 3:**
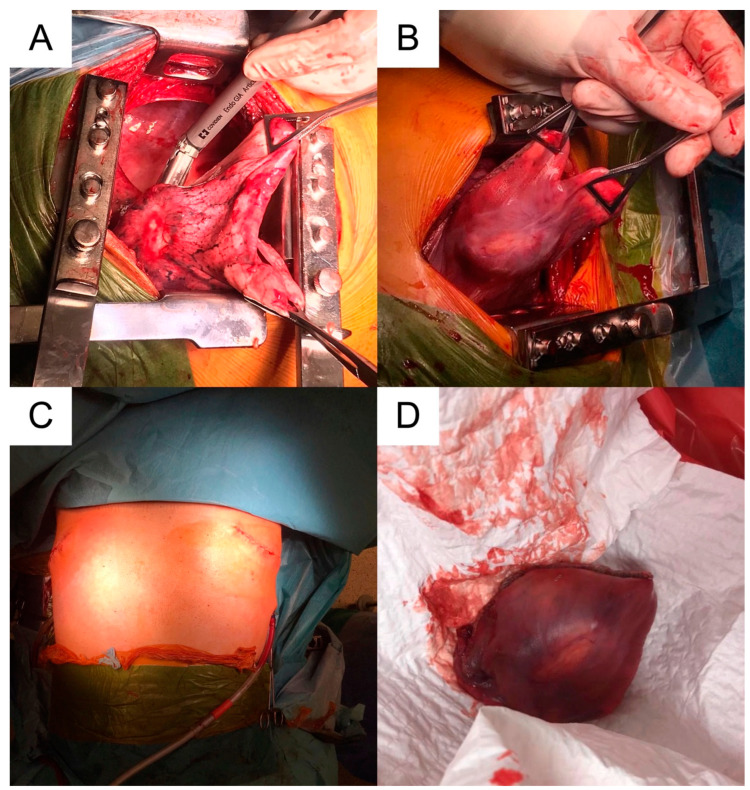
(**A**,**B**): Intraoperative snapshots of the wedge excisions; (**C**): prone position with simultaneous bilateral thoracotomies; (**D**): postoperative incisions and a typical specimen.

**Figure 4:**
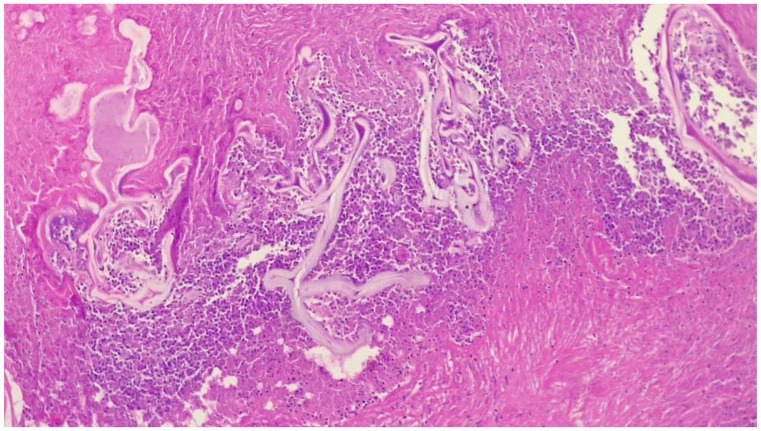
Histopathological specimen of case 1. Multiple cystic cuticular structures surrounded by abundant necrotic tissue and inflammatory reaction. Other areas showed in addition granulation tissue and fibrosis (H&E staining, 100×).

These were addressed synchronously with bilateral posterior thoracotomies in a prone position, using our experience in bilateral lung transplantation with this approach (left side: through the VII left intercostal space; right side: through the VI right intercostal space). Gentle manual palpation is necessary for localization. Wedge-type excisions were performed using standard automatic endostaplers of 45/60 mm length on clear macroscopic margins of minimum 1 cm interval (Figure 3). Histopathological confirmed extensive tissue necrosis and echinococcal structures (Figure 4). The parasite lesions may extend directly across the diaphragm and their rupture is a respiratory emergency.

**Figure 5:**
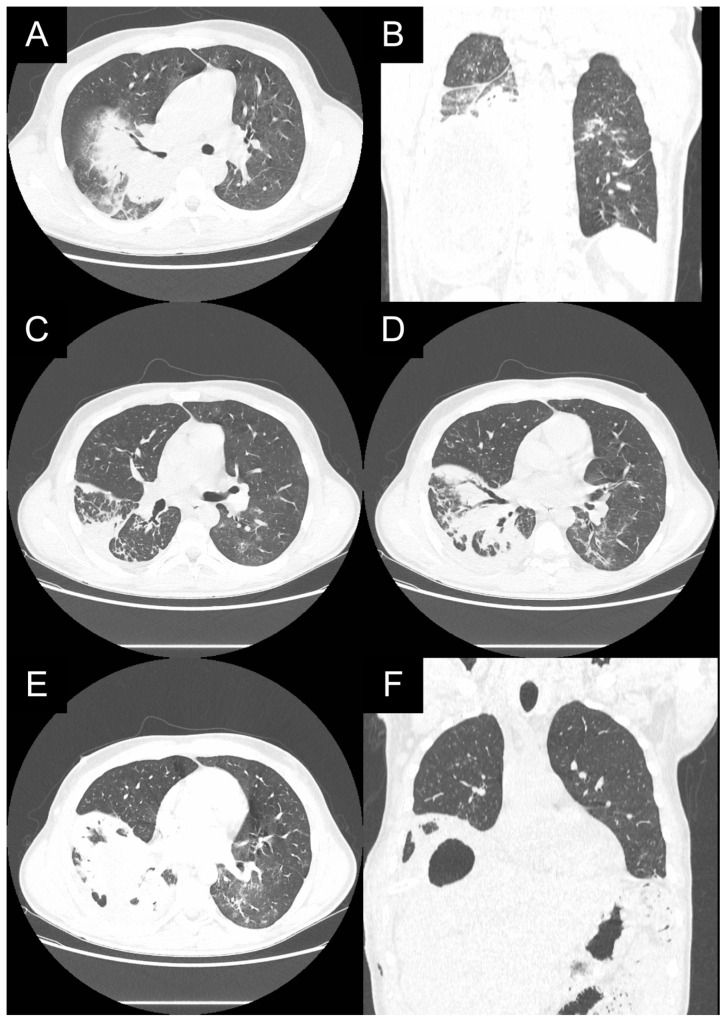
(**A**–**F**): A chest CT of patient 1 showing ruptured continuity of the hepatic lesion. The right lower lobe bronchi are filled with fluid from hepatic lesion. At bronchoscopy, an S10 fistula was detected.

**Figure 6:**
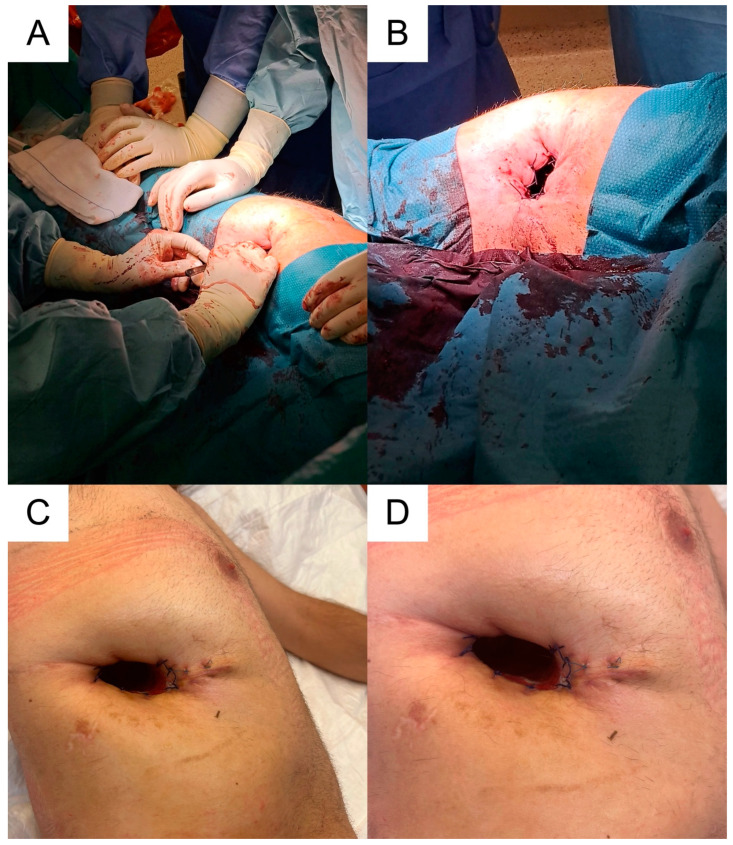
(**A**,**B**): Intraoperative images of open window thoracostomy (performed through the partial resection of the 9th right rib); (**C**,**D**): postoperative images of the open window thoracostomy during a dressing change.

**Figure 7:**
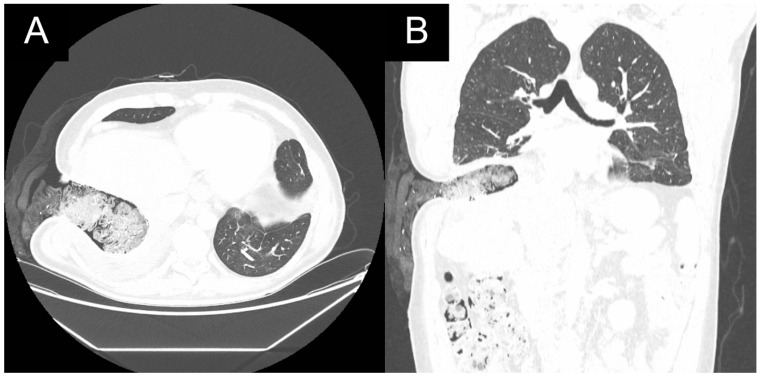
Postoperative chest CT in (**A**): axial and (**B**): coronal views of patient 1 with open window thoracostomy.

In the presented case, the hepatic lesion ruptured directly to the right lower segmental bronchus S10 as confirmed acutely in a flexible bronchofibersocopy, where large amounts of purulent discharge were lavaged. Chest CT showed communication of the ruptured lesion with the large right airways (Figure 5). This acute respiratory situation required an urgent surgical procedure. The presence of pneumonitis precludes parenchymal resections. A thoracotomy for exploration and meticulous debridement was followed by formation of an open fenestration at the level of the 9th rib to control infection without parenchymal resection (Figure 6). Regular dressing changes and long-term anthelminthics controlled the infection (Figure 7).

### 2.2. Case 2

**Figure 8:**
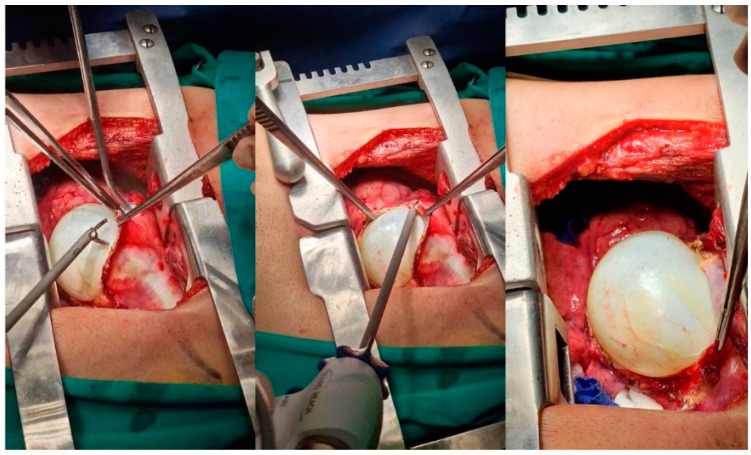
Intraoperative images of patient 2 with pulmonary echinococcosis. An energy device performs dissection without rupture of a large intraparenchymal cyst.

**Figure 9:**
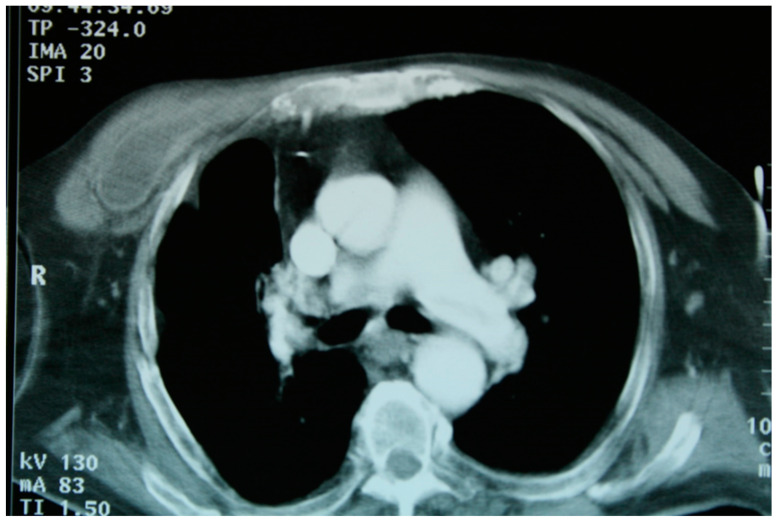
A chest CT of patient 2. A lesion misdiagnosed as a soft-tissue abscess that was excised in a similar manner as the previous case.

The uncomplicated large intraparenchymal pulmonary lesion. Typical diameters can exceed 15 cm. In such cases, wedge-excisions are unsuitable due to the size and a potentially central location. Instead, enucleation is indicated to spare parenchyma. The dissection of the cyst from the surrounding parenchyma is facilitated with a standard thoracoscopic energy device with ‘coagulate and cut’ functions and dissecting maneuvers, similar to adhesion lysis (Figure 8). Careful movements are required to avoid any rupture or spillage. Open bronchi should be recognized and ligated with nylon sutures 3-0 or 4-0 to prevent prolonged air leaks. Rarely, extra thoracic cysts may be misdiagnosed as soft tissue abscesses and percutaneously drained. Similarly, these cysts can be dissected free from the surroundings with an energy device (Figure 9).

### 2.3. Case 3

**Figure 10:**
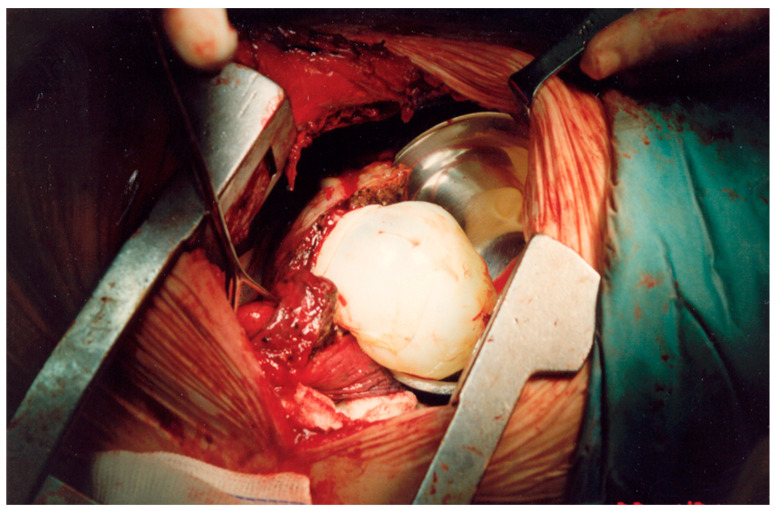
An intraoperative image of patient 3. For a large-sized cyst which is not suitable for excision by endostaplers, it is better to dissect it from the surrounding parenchyma, avoiding rupture to prevent anaphylaxis or reinfection. A collecting pot is preemptively next to the cyst to prevent accidental spillage of contents to the pleural cavity.

**Figure 11:**
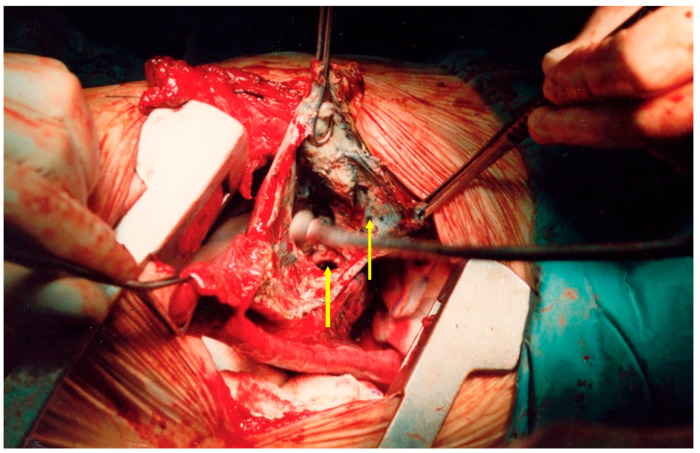
An intraoperative image of patient 3. Extensive dissection is required to remove the echinoccocal lesion and the surrounding necrotic tissue, leaving a raw parenchyma and frequently open segmental and subsegmental bronchi. Bronchial openings (yellow arrows) must be ligated for aerostasis. Endostaplers are not advised for this situation. We advise the use of absorbable sutures to ligate open ends and approximate raw surfaces.

The complicated large cyst. A large cyst that may rupture during dissection either when using a dissector or an energy device. In this unfortunate case, raw parenchymal surfaces may bleed, produce large air leaks, or leave redundant tissue. In this case, endostaplers or energy devices do not offer proven advantages. Instead, suturing (single or continuous, linear, or circular) should eliminate the space after careful debridement (Figure 10 and Figure 11).

## 3. Discussion

### 3.1. Characteristics of Echinococcus granulosus and Echinococcus multilocularis

#### 3.1.1. *Echinococcus granulosus*

Infection of *E. granulosus* is considered endemic in pastoral regions, such as Argentina, Chile, Central Asia, Africa, and Mediterranean countries. Single-organ involvement is a more frequent manifestation with the liver being the most common location (approximately 70%). Lung involvement is observed in 20% to 30% of CE patients [4]. Nevertheless, the parasite may form cysts in any human organ, including the brain, kidney, eye, and pancreas, among others [5]. The parasite breaks through the intestinal wall and migrates to the liver by portal circulation. However, embryos smaller than 0.3 mm can omit the liver and get to the pulmonary circulation through the hepatic vein and right heart. Furthermore, if the larvae get to the lymphatic system of the small intestine, they can enter the thoracic duct and migrate to the lungs through the internal jugular vein [6]. The majority of pulmonary CE lesions are unilateral. Furthermore, simultaneous involvement of the liver and lung is uncommon. According to a study by Khan et al., only 1.19% of cysts were in these two organs [7]. In a separate study by Joanny et al., the liver and lung were involved in 0.89% of CE cases [8]. In contrast, an older study by Dogan and colleagues demonstrated that simultaneous involvement of the lung and liver occurs in approximately 10% of cases in a cohort of 1055 patients with hydatidosis treated surgically [9]. Furthermore, CE may develop in more than two organs, but these cases occur extremely rarely [10]. Clinical course is usually asymptomatic, until the lesion starts to compress surrounding structures or the cyst ruptures [8].

#### 3.1.2. *Echinococcus multilocularis*

On the contrary to the CE, alveolar echinococcosis, caused by the *E. multilocularis,* is an endemic infection in North America, Central Europe, and East Asia. The parasite forms primary cysts almost exclusively in the liver. The infection with *E. multilocularis* resembles a malignant disease. It may be asymptomatic for up to 15 years of incubation. However, rapid growth of AE lesions has been identified in immunosuppressed patients, as demonstrated in reports of infected patients after solid organ transplantations [11,12]. Subsequently, the patient may present fatigue, weight loss, jaundice, or abdominal pain. The parasite invades surrounding structures and induces tissue necrosis. Furthermore, *E. multilocularis* may give metastasis to other organs [13]. According to a study by Aydin et al., 13% of AE cases developed pulmonary metastasis. The authors pointed at two routes through which the parasite may spread hematogenous, occurring in 73.5%, and transdiaphragmatic, which could be observed in 8.8% of included patients. However, in 17.7% of patients, both routes were identified. Moreover, in 55.9% of the patients with pulmonary involvement, the lesions were located bilaterally [14]. Therefore, similarly to the TNM staging system used in cancer, PNM staging is used in AE, where P indicates primary liver location, N describes if the parasite invades surrounding structures, and M is for metastasis [15]. Interestingly, a recent report by Fronhoffs and colleagues showed that AE might also mimic primary sclerosing cholangitis, which was discovered after liver transplantation [16]. AE is a progressive disease and, according to Ammann and Eckert, up to 90% of untreated patients die within 10 years from the beginning of symptoms [17]. However, due to significant advances in the treatment, a major improvement in prognosis has been observed since the 1970s [18].

### 3.2. Radiological Imaging

In pulmonary pathologies, X-ray is usually a primary method of investigation. Since pulmonary echinococcosis may be asymptomatic for a long time, the parasitic cysts are often diagnosed incidentally. CT is usually used to evaluate abnormal lesions detected in chest X-ray. According to a study by Wu et al., pulmonary CE lesions are usually single (60.9%) with an average size of 7.9 cm. The authors suggest that the loose pulmonary tissue and rich circulation may contribute to the fast growth of the cysts [19]. On the contrary, in AE, multiple lesions are observed more often. Furthermore, these lesions are irregular and manifest as scattered nodules [20]. However, a complicated cyst (ruptured, infected) may resemble other pathologies on imaging, such as lung abscess or cancer. In such cases, Choh et al. suggest adding T2-weighted MRI to the diagnosis process [21]. In rare cases of pleural hydatidosis, the infection may manifest as a massive pleural effusion, which is frequently of malignant origin [22]. Interestingly, pulmonary AE infection may also manifest as miliary metastases [23].

### 3.3. Other Diagnosis Procedures

Despite clinical course and radiological imaging, serology, histopathology, and molecular investigations may help in differentiating between CE and AE. Serological investigations often involve the detection of specific IgG antibodies [24]. Furthermore, searching for serum or hydatid fluid antigens is a useful method in the diagnosis process [25]. In CE, these antigens include hydatid protoscolices antigen (HPA), B antigen, B-rich antigen solution (BRAS), and crude antigen (CA) [26]. Furthermore, a broad range of antigens has also been identified in the case of AE, such as Em2, Em492, and Em18 [27]. Histopathological examination may provide beneficial information for a definite diagnosis. Alveolar lesion is characterized by a thin laminated layer and central necrosis. Moreover, calcifications within the areas of necrosis might be present. In contrast, CE is associated with a thick laminated layer and lack of necrosis. In addition, AE lesions are poorly delineated from surrounding tissue, while hydatid cysts are within fibrous capsule. Periodic acid–Schiff (PAS) staining should be performed in the differentiation process [28]. Furthermore, Barth and colleagues demonstrated that immunohistochemical staining with the Em2G11 monoclonal antibody allows for a diagnosis of AE [29]. Moreover, the expression of programmed cell death protein ligand 1 (PD-L1) in surgical specimens might indicate the postoperative outcomes in liver AE lesions [30]. Interestingly, Bellanger and colleagues showed that surgical treatment of the liver AE patients results in a reduction of serologic PD-L1 levels [31]. Molecular methods have also become more common in the diagnosis process. For instance, they are important in patients with AE lesions in atypical locations. Polymerase chain reaction (PCR) is a useful method, which can be implemented in the examinations of fluids, postoperative specimens, or biopsies. Knapp and colleagues point at several potential markers in patients with suspected echinococcosis, such as 16S-84 bp for AE and Trachsel-117 bp for CE [32].

### 3.4. Treatment

Currently, there are three therapeutic strategies for hepatic CE: surgery, ultrasound-guided aspiration, and chemotherapy [33,34]. Watch and wait strategy is usually implemented in case of inactive, asymptomatic cysts. Surgical resection remains the gold standard of treatment and might be a crucial therapeutic option. However, chemotherapy, PAIR (percutaneous aspiration, injection of chemicals, and respiration), or a cyst puncture might also be performed. According to the WHO, chemotherapy is used in inoperable tumors located primarily in the liver or lungs, as well as for patients with multiple cysts in at least two organs. It cannot be applied in patients with large or calcified cysts with a high risk of rupture [35]. Nevertheless, it needs to be highlighted that the non-invasive treatment might also be a beneficial supplementary therapy. Albendazole and mebendazole, which belong to the benzimidazole group, are used in prevention of a secondary echinococcosis, postoperative recurrence [36], or in patients with inoperable primary liver or lung echinococcosis [37]. Perioperative treatment with the use of benzimidazoles has also been reported to reduce intracystic pressure. The aim of this treatment is to soften the cysts and to simplify their removal during surgery [38]. PAIR is a less invasive approach, and it is correlated with a lower risk of complications than surgery [39] Percutaneous drainage with simultaneous albendazole therapy might also be an effective alternative for patients with uncomplicated liver echinococcosis [40]. In addition, combined therapy composed of albendazole with praziquantel may have an increased efficacy towards the parasitic cysts [34]. Currently, new therapeutic agents effective against *Echinococcus* are required. Several studies suggest that a variety of long known drugs might inhibit the growth of the parasite. These agents include mefloquine, pyronaridine, atovaquone, 5-fluorouracil, and metformin, among others [33]. Interestingly, Wang et al. investigated the PD-1/PD-L1 axis inhibition in mice with AE. The authors found that the use of anti-PD-L1 monoclonal antibodies decreased the parasite load and the number of liver lesions [41].

In pulmonary lesions, surgery aims to remove the parasite, prevent intraoperative dissemination, and preserve pulmonary parenchyma. Kavukcu et al. reviewed 1118 surgeries for pulmonary hydatidosis. Posterolateral thoracotomy was performed in 98.3% of patients. On the other hand, only 3.3% of patients underwent two-stage bilateral thoracotomy. Furthermore, only 3.3% of patients developed hydatid recurrence. Empyema was diagnosed in 0.3% of patients and these patients were treated with antibiotics and drainage [42]. Aydin et al. reviewed 170 consecutive surgeries of 153 patients with pulmonary hydatidosis. In the vast majority (96.5%) of cases, cystotomy and capitonnage was performed. Thoracoscopic wedge resections covered only 2.9% of all surgeries. A left upper lobectomy was performed in just one patient. Overall, empyema occurred in 1.3% of cases and recurrence developed in one patient [43]. Due to significant advances in minimally invasive procedures, several authors suggest using video-assisted thoracoscopic surgeries (VATS) alone or in combination with thoracotomy for resection of pulmonary hydatids [44,45]. Pulmonary cysts often develop in the right lower lobe. Therefore, resection of a pulmonary nodule through a laparotomy and transdiaphragmatic approach has also been described in the literature [46]. Interestingly, accumulating recent research is focused on developing vaccines against *E. granulosus* and *E. multilocularis* infections [47,48,49].

Despite advances and measures for zoonosis prevention and treatment, PE remains a clinical reality. Thoracic surgeons in non-endemic echinococcosis areas lack the experience in established procedures suitable for managing PE such as cystotomy, enucleation, capitonnage, or atypical resections, which occasionally are in combination with hepatic procedures [50,51]. If experience in these is lacking, surgeons may hesitate treating PE patients. However, individual cases of PE may require nothing more than everyday surgical skills identical to tumor surgery. The utilization of modern devices is greatly underdescribed in surgery for thoracic infections despite the fact that these facilitate much of the work. Stapling devices reduce operative time and secure aero- and hemostasis. Energy devices facilitate bloodless dissection and effective separation of cysts from parenchyma. There arises the question of what and when to use and how safe is it. We suggest that peripheral hydatid cysts up to 5 cm can be wedged out by (usually more than two) standard parenchymal endostapler cartidges (45 or 60 mm) in a linear or triangular fashion. This can be both a diagnostic and curative procedure. Dissection and enucleation with an aftermarket energy device is suitable for larger lesions. Cystotomy should be avoided to prevent spillage. More central large lesions may require lobar resection using a combination of the above instruments. Complex lesions with abdominal connections pleural fistulas or any situation where a sterile intrapleural cavity is not secured at the end of the procedure can be treated similar to a complex thoracic empyema with debridement and potentially with an open window thoracostomy for easy access and routine toileting. Other more complex PE cases should be treated in centers with experience in thoracic infections of surgical interest as their treatment should be well planned. A formal consultation with the infectious diseases team is of paramount importance to prevent recurrence as is the strict follow-up with laboratory and imaging investigations. The above should encourage thoracic surgeons to accept referrals of echinococcosis patients.

### 3.5. Differential Diagnosis

Due to non-specific symptoms and various manifestations in radiological imaging, there is a broad differential diagnosis for pulmonary echinococcosis. To begin with, tumors found on imaging in heavy smokers raise the suspicion of pulmonary neoplastic disease [52]. Furthermore, AE infection may induce typical cancer-related symptoms, which also suggest a metastatic disease [53]. Interestingly, Karamustafaoglu et al. described a patient with pulmonary hydatic cyst mimicking a Pancoast tumor. The reported patient experienced pain in the right arm and shoulder, together with arm paresthesia [54]. Furthermore, elevated standardized uptake value on the PET scan, which might accompany ruptured lesions, also points to malignancy [55]. Moreover, pulmonary echinococcosis in patients with a previous history of cancer suggests the dissemination of a neoplastic disease [56]. Furthermore, radiological examination may suggest the presence of other benign and rare pulmonary pathologies, such as benign metastasizing leiomyomas [57,58]. Additionally, simultaneous infections represent another diagnostic challenge. Few reports demonstrated the coexistence of echinococcosis with tuberculosis or aspergillosis [59,60,61].

## 4. Conclusions

Pulmonary echinococcosis is an uncommon but significant clinical entity. Differentiating between CE and AE is necessary for proper patient management and various methods may help in distinguishing between these two infections. In the presented cases, all the preoperative diagnostic workup has been completed by the referring internal medicine departments following the existing institutional guidelines valid at that time. Patients’ origin (case 1: Poland, cases 2 and 3: Greece), clinical course, radiological imaging, and intraoperative images suggest that the first patient was infected with *E. multilocularis*, while patients 2 and 3 with *E. granulosus.* Furthermore, histopathological examination of echinococcal lesion of the first patient revealed central necrosis, granulation tissue, and infiltration with neutrophils and lymphocytes, which points to alveolar variant of the infection. Patients with pulmonary echinococcosis may present symptoms such as cough, dyspnea, chest pain, haemoptysis, and fever, among others. Furthermore, radiological imaging may present solitary or multiple round lesions. As a result, the manifestation of pulmonary echinococcosis may resemble a primary or metastatic malignant disease, especially in patients with previous neoplasms or in heavy smokers. Furthermore, radiological examination may suggest the presence of other benign and rare pulmonary pathologies. The differential diagnosis of such lung tumors should include PE. Simple pulmonary echinococcal lesions up to 5 cm can be excised by endostaplers both for diagnostic and curative reasons. Larger cysts can be excised by energy devices unless large bronchial air leaks occur. Lobectomy may be required if a cyst is too large or located centrally. Complicated cysts require treatment by more extensive techniques. Inexperienced surgeons should not abstain but should carefully decide preoperatively how to proceed.

## Data Availability

Not applicable.
